# Lifespan models of athlete development: What have we learned from previous attempts?

**DOI:** 10.3389/fspor.2023.1179767

**Published:** 2023-03-31

**Authors:** Joseph Baker, Amy Gayman, Kathryn Johnston

**Affiliations:** ^1^ School of Kinesiology and Health Science, York University, Toronto, ON, Canada; ^2^ Department of Kinesiology and Physical Education, Wilfrid Laurier University, Waterloo, ON, Canada

**Keywords:** sport, aging, skill acquisition, models, frameworks, recreational sport, competitive sport

## Abstract

Sport has a unique place in many cultures, emphasizing the links between physical elements of movement with psychological and social outcomes. Sport participation continues to attract the interest of researchers from a range of perspectives, yet there remains a strong need to understand the “who”, “what”, “where”, “when” and “why” aspects of sport involvement over the life course. While the research literature includes multiple athlete development models that consider these components, they are incomplete frameworks for understanding lifespan sport engagement. In this article, we discuss the value in building multidimensional developmental models of sport participation that encapsulate experiences across all ages and stages of competitive and recreational sport, and pay special attention to the high degree of complexity of the movement between and within sport both competitively and recreationally. In addition, we highlight several challenges to creating such a lifespan development model, and consider areas of future direction to overcome some of these hurdles.

## Introduction

Sport has a unique place in many cultures, emphasizing the links between physical elements of movement with psychological and social outcomes. Many nations have seen a shift towards increased participation in organized, high-performance sport at the youth, adolescent, early and late adulthood levels. Accompanying this shift has been an increase in funding from national sporting bodies to cultivate athletic “talent” (1) as well as increased research attention (2), showcasing the importance of understanding the costs and benefits of this shift. For example, several recent reviews have been completed on issues related to athlete development, ranging from early specialization [see (3)] and youth athletic development models [see (4)] to sport for older adults (5). In many sports, in many countries, participation can occur across the lifespan.

The concept of “lifespan development” has been used in domains such as education (6), employment (7), and medicine (8) to explore the mechanisms that generate commonalities, variability, and change in behaviour across the spectrum of human experience, from fetal development to old age (9, 10). Often, such research leads to the creation of conceptual models that help shape the way we interpret and predict behaviour. For example, modeling psychological development (i.e., psychological and neuronal changes and adaptations throughout the lifespan), allows exploration of complex person-environment interactions (11, 12). A more complete understanding subsequently informs interventions designed to support different types of learners (13, 14). While these models provide opportunities to tailor conceptually-supported interventions that promote optimal development, as well as evidence-informed support and instruction, caution has been raised in crafting models that are too reductionist and “nonrepresentative” of human experience, which may disproportionately affect some individuals or groups [c.f., (15, 16)].

In sport, lifespan models offer the same potential benefits. For example, they can provide insight into individual development in, and through, sport over the lifespan, spanning various ages, stages, abilities, and backgrounds, among other factors. This knowledge can also inform research (i.e., drive the creation and use of various methodologies and methods) and practice (i.e., including aspects of programming, techniques, strategies) for coaching and coach education [e.g., (17, 18)] as well as training and interventions (19, 20). In turn, lifespan models have the potential to enhance our understanding of how to foster more holistic (i.e., considering an athlete's psychological, physical, social, and spiritual needs), inclusive (i.e., reducing barriers for participation and success), and developmentally-appropriate sport programs that cater to athlete needs and promote sustained sport involvement, improved performance, and health across the lifespan.

**Figure 1:**
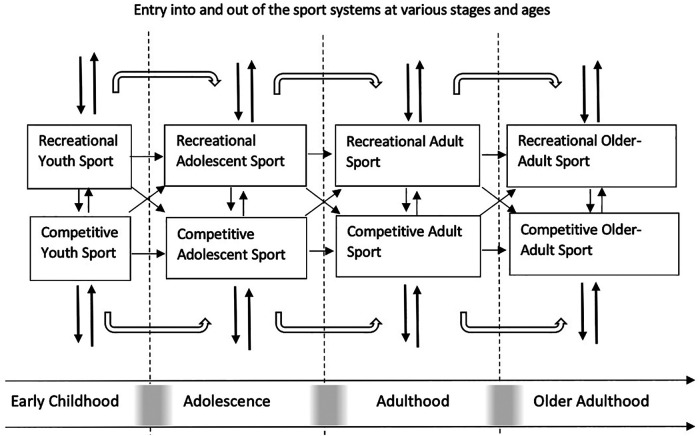
A proposed model of the complex and intertwined pathways of sport experience across the lifespan. The thick arrows at each stage from youth, adolescents, adulthood and older adulthood indicate that a person can enter (and exit) at various ages and stages into either competitive sport or recreational sport. The white arrow with the black outline indicates the opportunity for re-entry into the sport system once someone has left a sport. This could be re-entry into that same sport or re-entry into different sports. The small, short arrows indicate the movement between and within the recreational and competitive sport systems. What is further depicted is the lifespan stages of development sub-divided into the 4 broad stages examined in prior sport models, early childhood, adolescence, adulthood, and older adulthood, which will allow for age-and development-stage specifications/recommendations/interventions for sport participation, while also appreciating there is no discrete timeline when someone ends one stage and starts another, as indicated by the transition “grey areas” between each stage that represent individual variation of developmental age and stage.

Current discussions of athlete development are dominated by the Developmental Model of Sport Participation [DSMP: (21)] and the Long-Term Athlete Development (LTAD) framework (22) as well as emerging models like Australia's Foundations, Talent, Expertise and Mastery (FTEM) framework (23), and Lloyd and Oliver's (24) Youth Physical Development Model [see (25) for a review]. A common thread connecting these areas of exploration is their focus on athletes in the early stages of development (pre-elite). This has left little work examining elite athlete development [i.e., at the highest level; (26)] or post-elite development (i.e., the period after the high-performance career has ended). One example of a model that has worked to incorporate both the elite and post-elite developmental stages is Canada's Sport for Life framework, which highlights the potential value of capturing sport participation over the lifespan (both recreationally and competitively from youth to older adulthood; (27)). Models such as this, which span broader developmental periods into older adulthood, are becoming increasingly more relevant, as recent evidence on Masters sport (i.e., international-level competition for individuals over the age of 35^1^) indicates the number of international competitors (including the number of nations participating; currently over 50) is at an all time high (see https://imga.ch/ for more). This presents a critical opportunity for researchers and practitioners to investigate adult and older-adult populations' experiences in sport from a biopsychosocial perspective—with the hope of building more inclusive, accessible, and developmentally-appropriate programs for sport involvement across the lifespan. Ultimately, a comprehensive model of lifespan athlete development would go beyond simply stating broad goals such as being “active” or “competitive for life”. Instead, it would be capable of providing knowledge of, and support and guidance for, phases of athlete development at all levels of involvement across this extended period, as well as transitions out of, or between stages. Moreover, given the obvious inter-connections between participatory and high-performance sport, a thorough model would denote the various pathways through the sports system and acknowledge the complex interactions between competitive and recreational sport participation, and how these different forms of sport engagement interact to shape lifespan sport involvement (Figure 1).

## Challenges and concerns in creating a lifespan model of athlete development

By no means do we wish to imply the creation of this type of model would be easy. Like other lifespan development models, sport development models come with some obvious and less-obvious challenges. In the following section, we briefly highlight several key challenges for future work.

### Language clarity

One of the most fundamental concerns raised by researchers and practitioners pertains to the clarity and consistency of language. Several recent papers have emphasized the inconsistent and “blurry” nature of terminology in athlete development research. For instance, the DSMP regularly refers to the notions of “sampling” and “early specialization”. These terms have received recent attention by the research community [see (3, 28), respectively], highlighting their conceptual confusion and lack of clarity. The absence of an agreed upon definition of what these terms [and other terms; see for example, (29)] mean, will continue to make measurement, assessment, and implementation strategies imprecise and difficult.

Perhaps the most glaring discrepancy relates to a broad, yet foundational component of these models—the conceptualization of “sport”. Despite the widespread use of sport as a general term, it is difficult to pin down a clear definition (30). Furthermore, research and popular discourse tell us that sport is not just one “thing”; rather, there are noteworthy variabilities across countries and cultures. With emerging technologies and interests, coupled with the growth of new sport disciplines (e.g., e-sports, pickleball, and disc golf), these definitional lines may become even less clear. This ambiguity further complicates the creation and validation of lifespan models, which need to accommodate such definitions. As growth and expansion in the types of sports available continues, a comprehensive model will need to consider and evolve to capture inter-sport differences (e.g., age of entry, specialization, and peak performance) and variability across demographic groups (e.g., male vs. female sports, youth sport compared to Masters sport, Olympic vs. Paralympic sports, amateur compared to professional).

### Development as a lifespan process

A comprehensive model also needs to reflect development as a lifelong process, integrating elements of learning, expertise development, and competitive performance/success across the life course. A key learning from prior work is the need to create “optimized training environments” that match the learning environment to the needs of the athlete's stage of development (e.g., based on maturity, experience, etc.). Moreover, learning, skill acquisition and performance needs will undoubtedly change across development, although precisely *when* and *how* remain largely unknown (see below for more detail on this point).

 A greater understanding of the processes and predictors of athlete development across the lifespan, with appreciation of the complexities involved at different life stages, has the potential to inform and strengthen public health policies and priorities. In this sense, a clearer understanding of how individuals experience various stages of development over time could support the creation of more effective, inclusive, and developmentally appropriate programs and interventions.

### Integrating related aspects of athlete development

Currently, the conceptualization of athlete development is convoluted by the broad range of related topics under study (e.g., athletic development, career transitions, participant development, positive youth development, talent development), which vary in terms of their emphasis on personal growth, lifelong sport involvement, sport-specific expertise, and performance excellence. The often disconnected and narrow focus of different approaches to understanding the development of sport participants has prompted calls for greater interdisciplinary collaboration and knowledge sharing (31–34).

Ultimately, future efforts will need to adopt a multidisciplinary lens that accounts for the holistic, integrated nature of athlete development. Researchers have recognized that development is ongoing and dynamic. It is a complex phenomenon influenced by a host of factors inherent to person-environment interactions within and beyond the sport setting (35, 36). Moreover, athlete development rarely unfolds in a linear manner. It is a highly individualized process with many different participation pathways and career transitions to consider (33, 34, 37, 38). Furthermore, performance success at the highest level of competitive or professional sport is not the final developmental stage or end goal for the majority of sport participants who pursue recreational, community-based forms of engagement. An inclusive framework is needed to gain insight into the factors that support and constrain enhanced biopsychosocial development as individuals of all ages, involved in all levels and contexts of sport, move in and out of the sport system over time (38).

## Into the unknown: Key questions for future work

### Do we understand the purpose(s) of sport across the lifespan?

Adding further complexity to the issues discussed above, is the reality that sport holds different, often conflicting, meanings and purposes across the lifespan. In childhood and youth, the goals of youth sport have been framed as relating to participation, performance, and personal development (39), although presumably the first is the mechanism driving effects in the latter two. From this perspective, the value of sport is in its potential to promote positive youth development (40) and the acquisition of fundamental movement skills and physical literacy (41). However, the purpose(s) of sport later in life is less clear. Some researchers have suggested it is valuable for challenging negative age-related stereotypes (42), decreasing chronic disease burden (43), and promoting positive developmental outcomes (44), as well as more obvious outcomes such as enjoyment and social connection.

Importantly, it will be critical to distinguish the value(s) and benefits of sport in later life compared to neighboring domains like “exercise” and “physical activity”, which are commonly promoted at this stage of life. Without a clear understanding of the role and value of sport across the lifespan, and if/when/how the objectives of sport change during different life stages, it is difficult to understand how to enhance an athlete's development, regardless of what the objective of that engagement might be.

### What do “pathways” for older athletes look like?

Evidence-based perspectives of developmental trajectories in middle-to late-adulthood are another important consideration for future research on the role of sport for older people. Although some models of sport involvement (e.g., LTAD and FTEM) recognize participation occurs across the lifespan, engagement is usually presented in generic ways such as in Canada's Sport for Life Model which defines the entire phase after sporting excellence as simply active, fit or competitive for life, based on the type of engagement. Longitudinal research examining all levels and patterns of participation, including athletes who dropout or withdraw from sport, is sorely needed (45).

Importantly, the literature pertaining to sport for older people has focused predominantly on competitive athletes involved in Masters and Senior Games with much less attention devoted to individuals who participate in community-based recreational sport (42). We know relatively little about the different combinations of pathways later in life that adults may pursue, what factors influence participation patterns and developmental opportunities from childhood to older adulthood, and best/better practices to support the developmental goals of middle-aged and older sport participants. To gain insight into patterns of stability and instability in developmental trajectories over time, researchers need longitudinal data pertaining to sport involvement of diverse samples as they age, to assess continued, resumed, and first-time involvement in sport (46).

### One model or several?

A relatively indisputable finding from previous work is that sport is highly nuanced (i.e., varying across types, age groups, competition levels, cultures, and time). From this perspective, it may be too much to expect a single model to adequately capture the variability in what sport is and means for all individuals across all these contexts. Potentially, a general model could dilute attention to the critical issues for athlete development in a single sport. One example can be seen in how the issue of “early specialization” has been framed in general models—as something to be avoided, unless you happen to be in a sport that specializes early (e.g., gymnastics).

Instead, it might be useful to reframe the overall purpose of athlete development models/frameworks to providing insight and recommendations for different categories of sports. Returning to our example of early specialization noted above, an athlete development model for “early specialization sports” (or, perhaps a more precise category name would be “sports with an earlier age of peak performance” or “aesthetic sports”) such as gymnastics, diving, and figure skating would allow a more thoughtful discussion of the risks and consequences of an athlete specializing early so that these can be managed by coaches and practitioners. Other categories may also be helpful, allowing policy makers, administrators, and coaches to focus on the unique needs of athletes in similar contexts, such as categories of Paralympic sports (47), low participation sports [e.g., see (48) for a discussion of this in Dutch table tennis], women's sport (49), and/or Masters sport (17).

While these concerns are warranted, we believe there is still value in a general lifespan model of athlete development—provided it focused on elements best captured in a generic model. For example, this model may be most useful as a guiding framework, reflecting knowledge about human development broadly (e.g., what is appropriate for a given age group?). As we learn more about the types of training and experiences best suited for different participation outcomes (e.g., lifespan participation, recreational competition, or elite athlete development), this general foundation may provide guidance for advocating one form of training over another. For instance, satisfying basic psychological needs of social connection and autonomy may be more conducive to recreational or life-long participation, while a focus on developing feelings of competence and performance-focused orientations may be more strongly related to elite skill development. This general model could inform context specific models that focus on elements related to different categories of sport. Potentially, these category models could be followed up with sport specific ones. For instance, a sport may consider issues relative to their specific sport context (e.g., differences in performance requirements, training resources, etc.), how these relate to other sports within the same category (e.g., are there ways to share resources to improve system efficiency?), and whether athlete development decisions correspond to broad learning and developmental needs to individuals at that stage of human development. This *Sport-Category-General* approach may alleviate some of the criticisms that have been made of general models in the past.

## Concluding thoughts

A comprehensive approach is needed to understand *how* and *why* to promote better supported athlete development from childhood to older adulthood. A lifespan model(s) of athlete development could guide empirical investigations of personal and environment factors that shape biopsychosocial development over time as sport participants age, and account for varying motives, goals, and participation patterns. We recognize, however, that such efforts may be affected by wider environmental, cultural, and political issues that shape program development, applied practice, policy implementation, and sport governance. Pragmatically, the adoption and implementation of a lifespan model in the applied setting may hinge on the value key stakeholders and society, in general, ascribe to sport as a context to facilitate athlete development beyond youth, high performance competitive sport. Perhaps most notably, successful creation and implementation of a comprehensive, evidence-informed, general model of lifespan athlete development will be driven by an integrated, collegial, and collaborative approach among researchers, applied scientists, coaches, and policy makers. Although this may be a difficult hill to climb, there are undoubtedly riches on the other side.

## Footnotes

^1^The youngest participants are 22 for sports like gymnastics that have an earlier peak-age.

## References

[1] Weissensteiner J. The global evolution of talent promotion within Olympic sports: a focus on the national systems and contribution of the former German Democratic Republic, Australia and the United Kingdom. Front Sports Act Living. (2023) 4:1124234. 10.3389/fspor.2022.1124234 36819734 PMC9934925

[2] Baker J Wilson S Johnston K Dehghansai N Koenigsberg A De Vegt S Talent research in sport 1990–2018: a scoping review. Front Psychol. (2020) 11:607710. 10.3389/fpsyg.2020.607710 33324305 PMC7723867

[3] Mosher A Fraser-Thomas J Baker J. What defines early specialization: a systematic review of literature. Front Sports Act Living. (2020) 27(2):596229. 10.3389/fspor.2020.596229 PMC7739675 33345176

[4] Varghese M Ruparell S LaBella C. Youth athlete development models: a narrative review. Sports Health. (2022) 14(1):20–9. 10.1177/1941738121105539 34758649 PMC8669922

[5] Patelia S Mazhar A Baker J. What do we know about the value of sport for older adults: a scoping review. J Aging Phys Act. (2023) 1:1–16. 10.1123/japa.2022-0146 36669504

[6] Alexander PA. The path to competence: a lifespan developmental perspective on reading. J Lit Res. (2005) 37(4):413–36. 10.1207/s15548430jlr3704_1

[7] Rudolph CW Zacher H. Considering generations from a lifespan developmental perspective. Work Aging Retirement. (2017) 3(2):113–29. 10.1093/workar/waw019

[8] Walco GA Krane EJ Schmader KE Weiner DK. Applying a lifespan developmental perspective to chronic pain: pediatrics to geriatrics. J Pain. (2016) 17(9):T108–17. 10.1016/j.jpain.2015.11.003 27586828

[9] Lindenberger U Lövdén M. Brain plasticity in human lifespan development: the exploration–selection–refinement model. Ann Rev Dev Psychol. (2019) 1:197–222. 10.1146/annurev-devpsych-121318-085229

[10] Lindenberger U Li SC Bäckman L. Delineating brain-behavior mappings across the lifespan: substantive and methodological advances in developmental neuroscience. Editorial. Neurosci Biobehav Rev. (2006) 30(6):713–7. 10.1016/j.neubiorev.2006.06.011 16928401

[11] Krakauer JW Ghazanfar AA Gomez-Marin A MacIver MA Poeppel D. Neuroscience needs behavior: correcting a reductionist bias. Neuron. (2017) 93(3):480–90. 10.1016/j.neuron.2016.12.041 28182904

[12] Schaie KW. A field-theory approach to age changes in cognitive behavior. Vita Humana. (1962) 5(3):129–41. http://www.jstor.org/stable/26761552 13976649 10.1159/000269554

[13] Lövdén M Fratiglioni L Glymour MM Lindenberger U Tucker-Drob EM. Education and cognitive functioning across the life span. Psychol Sci Public Interest. (2020) 21(1):6–41. 10.1177/1529100620920576 32772803 PMC7425377

[14] Wenger E Kühn S Verrel J Mårtensson J Bodammer NC Lindenberger U Repeated structural imaging reveals nonlinear progression of experience-dependent volume changes in human motor cortex. Cereb Cortex. (2017) 27(5):2911–25. 10.1093/cercor/bhw141 27226440

[15] Gould SJ. A critique of Heckhausen and Schulz’s (1995) life-span theory of control from a cross-cultural perspective. Psychol Rev. (1999) 106(3):597–604. 10.1037/0033-295X.106.3.597

[16] Heckhausen J Schulz R. A life-span theory of control. Psychol Rev. (1995) 102(2):284–304. 10.1037/0033-295X.102.2.284 7740091

[17] Callary B Young B Rathwell S. Coaching masters athletes: Advancing research and practice in adult sport. New York: Routledge (2021).

[18] Lemyre F Trudel P Durand-Bush N. How youth-sport coaches learn to coach. Sport Psychol. (2007) 21(2):191–209. 10.1123/tsp.21.2.191

[19] Oh J Chopik WJ Konrath S Grimm KJ. Longitudinal changes in empathy across the life span in six samples of human development. Soc Psychol Personal Sci. (2020) 11(2):244–53. 10.1177/19485506198494

[20] Smart J. Disability across the developmental life span: For the rehabilitation counselor. New York: Springer (2011).

[21] Côté J Vierimaa M. The developmental model of sport participation: 15 years after its first conceptualization. Sci Sports. (2014) 29:S63–9. 10.1016/j.scispo.2014.08.133

[22] Balyi I Way R Higgs C. Long-term athlete development. Champaign: Human Kinetics (2013).

[23] Gulbin JP Croser MJ Morley EJ Weissensteiner JR. An integrated framework for the optimisation of sport and athlete development: a practitioner approach. J Sports Sci. (2013) 31(12):1319–31. 10.1080/02640414.2013.781661 23631711

[24] Lloyd RS Oliver JL. The youth physical development model: a new approach to long-term athletic development. Strength Cond J. (2012) 34(3):61–72. 10.1519/SSC.0b013e31825760ea

[25] Baker J Wattie N. Athlete development models. In: Gould D Mallett C, editors. Sport coaches’ handbook. Champaign: Human Kinetics (2020). p. 119–32.

[26] Baker J Koz D Kungl AM Fraser-Thomas J Schorer J. Staying at the top: playing position and performance affect career length in professional sport. High Ability Stud. (2013) 24(1):63–76. 10.1080/13598139.2012.738325

[27] Higgs C Way R Harber V Jurbala P Balyi I. Long-term development in sport and physical activity 3.0. Sport for Life. (2019).

[28] Murata A Goldman DE Martin LJ Turnnidge J Bruner MW Côté J. Sampling between sports and athlete development: a scoping review. Int J Sport Exerc Psychol. (2021) 30:1752–6. 10.1080/1612197X.2021.1995021

[29] McAuley AB Baker J Kelly AL. Defining “elite” status in sport: from chaos to clarity. German J Exerc Sport Res. (2022) 52(1):193. 10.1007/s12662-021-00737-3 PMC8340584 40477382

[30] Carlson C Frías FJL Schieman K Reid HL McClelland J Strudler K Defining sport: Conceptions and borderlines. Lanham: Lexington Books (2016).

[31] Bruner MW Erickson K Wilson B Côté J. An appraisal of athlete development models through citation network analysis. Psychol Sport Exerc. (2010) 11(2):133–9. 10.1016/j.psychsport.2009.05.008

[32] Collins D Toms M MacNamara A Ford PA. Rethinking participant development in sport and physical activity. In: Holt NL Talbot M, editors. Lifelong engagement in sport and physical activity: Participation and performance across the lifespan. Abingdon: Taylor & Francis (2011). p. 45–62.

[33] Coutinho P Mesquita I Fonseca AM. Talent development in sport: a critical review of pathways to expert performance. Sports Sci Coaching. (2016) 1(2):279–93. 10.1177/1747954116637499

[34] Stambulova NB Ryba TV Henriksen K. Career development and transitions of athletes: The international society of sport psychology position stand revisited. International Journal of Sport and Exercise Psychology. (2021) 19(4):524–550. 10.1080/1612197X.2020.1737836

[35] Abbott A Collins D. Eliminating the dichotomy between theory and practice in talent identification and development: considering the role of psychology. J Sports Sci. (2004) 22(5):395–408. 10.1080/02640410410001675324 15160593

[36] Baker J Wattie N Schorer J. A proposed conceptualization of talent in sport: the first step in a long and winding road. Psychol Sport Exerc. (2019) 43:27–33. 10.1016/j.psychsport.2018.12.016

[37] Baker J Johnston K Wojtowicz M Wattie N. What do we really know about elite athlete development? Limitations and gaps in current understanding. Br J Sports Med. (2022) 56(23):1331–2. 10.1136/bjsports-2022-105494 35973756

[38] Bergeron MF Mountjoy M Armstrong N Chia M Côté J Emery CA International Olympic committee consensus statement on youth athletic development. Br J Sports Med. (2015) 49:843–51. 10.1136/bjsports-2015-094962 26084524

[39] Côté J Strachan L Fraser-Thomas J. Participation, personal development and performance through youth sport. In: Holt N, editor. Positive youth development through sport. Abingdon: Routledge (2008). p. 34–45.

[40] Holt NL Deal CJ Pankow K. Positive youth development through sport. In: Tanenbaum G Eklund RC, editors. Handbook of sport psychology. 4th ed. Hoboken: Wiley (2020). p. 429–46.

[41] Corbin CB. Implications of physical literacy for research and practice: a commentary. Res Q Exerc Sport. (2016) 87(1):14–27. 10.1080/02701367.2016.1124722 26889581

[42] Jenkin CR Eime RM Westerbeek H O’Sullivan G van Uffelen JGZ. Sport and ageing: a systematic review of the determinants and trends of participation in sport for older adults. BMC Public Health. (2017) 17:976. 10.1186/s12889-017-4970-8 29273036 PMC5741887

[43] Patelia S Stone RC El-Bakri R Adli M Baker J. Masters or pawns? Examining injury and chronic disease in male Masters Athletes and chess players compared to population norms from the Canadian community health survey. Eur Rev Aging Phys Act. (2018) 15:1–9. 10.1186/s11556-018-0204-z 30519363 PMC6267924

[44] Baker J Fraser-Thomas J Dionigi R Horton S. Sport participation and positive development in older persons. Eur Rev Aging Phys Act. (2010) 7(1):3–12. 10.1007/s11556-009-0054-9

[45] Gallant F Bélanger M. Empirical support for the tenets of sport participation and physical activity-based models: a scoping review. Front Sports Act Living. (2021) 3:1–15. 10.3389/fspor.2021.741495 PMC8552970 34723180

[46] Gayman AM Fraser-Thomas J Baker J. Relational developmental systems metatheory: a conceptual framework to understand and promote older adults’ involvement in sport. Eur Rev Aging Phys Act. (2017) 14:12. 10.1186/s11556-017-0182-6 28770013 PMC5526265

[47] Dehghansai N Pinder RA Baker J. Talent development in paralympic sport. Abingdon: Routledge (2022).

[48] Faber I Damsma T Pion J. Finding talent and establishing the road to excellence in table tennis: the Dutch case. In: Baker J Cobley S Schorer J, editors. Talent identification and development in sport. 2nd ed. Abingdon: Routledge (2020). p. 115–29.

[49] MacMahon C Clarke A Leabeater A Roberts A. Female sport expertise through a skill acquisition lens. In: Farrow D Baker J MacMahon C, editors. Developing sports expertise: Researchers and coaches put theory into practice. 3rd ed. Abingdon: Routledge (in press).

